# Exploring COVID-19 Vaccine Decision Making: Insights from ‘One-Shot Wonders’ and ‘Booster Enthusiasts’

**DOI:** 10.3390/ijerph21081054

**Published:** 2024-08-12

**Authors:** Josefina Nuñez Sahr, Angela M. Parcesepe, William You, Denis Nash, Kate Penrose, Milton Leonard Wainberg, Subha Balasubramanian, Bai Xi Jasmine Chan, Rachael Piltch-Loeb

**Affiliations:** 1 Institute for Implementation Science in Population Health, City University of New York, New York, NY 10027, USA; 2 The Carolina Population Center, University of North Carolina at Chapel Hill, Chapel Hill, NC 27599, USA; 3 Department of Maternal and Child Health, Gillings School of Global Public Health, University of North Carolina at Chapel Hill, Chapel Hill, NC 27599, USA; 4 Department of Epidemiology and Biostatistics, Graduate School of Public Health and Health Policy, City University of New York, New York, NY 10027, USA; 5 Department of Psychiatry, Columbia University Vagelos College of Physicians and Surgeons/New York State Psychiatric Institute, New York, NY 10032, USA

**Keywords:** COVID-19, vaccines, health behavior, anxiety, depression, qualitative studies

## Abstract

Within the USA, the uptake of the updated COVID-19 vaccines is suboptimal despite health authority recommendations. This study used qualitative methods to examine factors influencing COVID-19 vaccine decision making and the effects of anxiety and depression on these decisions within the CHASING COVID Cohort (C3). Between October and December 2023, we conducted 25 interviews with participants from 16 different US states, 14 of whom endorsed recent symptoms of anxiety and/or depression. Using grounded theory methodology for coding and thematic analysis, we categorized participants into “One-Shot Wonders” and “Booster Enthusiasts”. Our findings indicate that the US COVID-19 vaccination environment has shifted from active promotion to a notable absence of COVID-19 discussions, leading to reduced worry about infection and severe illness, diminished perception of the benefits of the vaccine on personal and community levels, and fewer cues to action. Initially influential factors like family, personal experiences, and physician recommendations lost impact over time. Although the relationship between symptoms of depression and anxiety and vaccination was not prominent, one case highlighted a direct relationship. The study emphasizes the importance of timely and accurate public health messaging adaptable to individuals’ needs and misconceptions, highlighting the need for dynamic communication strategies in future initiatives with rapidly changing landscapes.

## 1. Introduction

The low uptake of the latest COVID-19 vaccines poses a global public health concern. Within the United States, vaccination for the updated 2023–2024 COVID-19 vaccine remains suboptimal. Despite 81.4% of the population receiving at least one dose of any COVID-19 vaccine since 2020, as of 4 May 2024, only 22.5% of adults and 14.1% of children under 17 years old had received the latest vaccine for the 2023–2024 season [1]. This trend is expected to continue, with 41.3% of US adults stating they probably or definitely will not get a COVID-19 vaccine as of July 2024 [2]. Limited uptake of COVID-19 boosters persists despite ongoing COVID-19 transmission, severe illness, and mortality—between September 2023 and January 2024, there was a steady increase in hospital admissions and deaths attributable to COVID-19, with weekly positivity rates fluctuating between 9 and 12% [3].

The uptake of COVID-19 vaccines varies across states, with rates ranging from 10.7% in Mississippi to 41.6% in Vermont as of May 2024 [1]. However, in the fall–winter period of 2023–2024, nearly all state health departments in the US continued to recommend COVID-19 vaccination to all people aged 6 months and older, adhering to recommendations from the Centers for Disease Control and Prevention (CDC) (Effective 1 September 2023, the Department of State Health Services of Texas was prohibited from using appropriated funds to promote or advertise COVID-19 vaccinations [4]. In Florida, the State Surgeon General recommended against the COVID-19 booster for individuals under 65 and states specific guidelines for those over 65 to consider before getting a vaccine [5]. There have been many theorized reasons as to why booster uptake has been low despite recommendations from federal, state, and local health authorities. The CDC points to a lack of healthcare provider recommendation, concerns or issues about unknown or serious side effects, having experienced mild side effects from prior COVID-19 vaccines, and lack of time or forgetting to get vaccinated [6]. Other studies indicate macrolevel factors, including trust in the healthcare system, public health authorities and governmental institutions, as well as group-level considerations that encompass age, sex, race/ethnicity, political affiliation, income, and educational attainment [7,8].

Researchers have also explored how social science frameworks can help better understand vaccine decision making. Specific constructs that may influence vaccine hesitancy and uptake include perception of the susceptibility to a disease, its severity, the barriers to preventive actions, and the perceived benefit of vaccination [9,10]. Other studies have taken a broader perspective, linking the multifaceted influences from intrapersonal, interpersonal, organizational, community, and public policy factors and significantly associating them with COVID-19 preventive behavior including vaccine uptake [11]. However, most studies attempt to quantify the effect of these factors over statements of vaccine hesitancy including vaccine safety and efficacy, and there have been few qualitative studies with a broad and diverse sample of US adults that have aimed to document influential factors in the decision-making process to actually receive a COVID-19 vaccine and vaccine booster.

Recently, some researchers have studied how the constant stream of information on COVID-19 preventive behaviors have led to message overload, contributing to both confusion, anxiety, and, possibly, inaction [12]. This goes hand in hand with recent findings from the Chasing COVID Cohort study, revealing that individuals experiencing moderate to severe symptoms of anxiety or depression had a lower likelihood of receiving additional COVID-19 vaccine doses [13]. However, the multifaceted challenges imposed by the pandemic, including prolonged social isolation, heightened reliance on technology, restrictions on mobility and travel, widespread job losses, economic instability, and pervasive uncertainty, have collectively contributed to a decline in individuals’ mental well-being [14,15,16], and research has identified an increased prevalence of symptoms of anxiety and depression [15,16,17]. In light of these findings, some studies that have highlighted mental health should be considered an axis of inequality for vaccine uptake [18]. All in all, this highlights the complex interplay between mental health and vaccination behaviors within the broader context of the pandemic’s impact on individuals’ perceptions and decision-making processes. Despite this, there is limited research exploring this issue from a qualitative perspective that captures the nuances and subjective experiences of individuals.

Despite extensive studies that have researched vaccine hesitancy since before the pandemic, there is limited understanding of why individuals who initially received the COVID-19 vaccine choose to forgo subsequent boosters. The potential influence of mental health symptoms on this decision remains unexplored, which is particularly relevant given the mental health crisis onset by the pandemic [14,15,16]. Although numerous studies have tested different theories and factors that may affect vaccine uptake, there is a scarcity of qualitative research using a grounded theory approach that allows participant responses to develop patterns and themes organically rather than being confined to pre-established theories. In the current endemic scenario, understanding why people are not continuing to receive COVID-19 boosters is a pivotal aspect of maintaining low infection rates and achieving long-term control over the virus.

Given the low level of booster uptake across the United States, coupled with limited understanding of the relationship between symptoms of anxiety and depression and vaccination, we took a grounded theory approach to exploring this in the Communities, Households and SARS-CoV-2 Epidemiology (CHASING) COVID Cohort Study, a community-based prospective cohort study launched during the upswing of the USA COVID-19 epidemic that has been systematically monitoring the incidence and determinants of SARS-CoV-2 outcomes, symptoms of anxiety and depression, and economic outcomes. Through a qualitative methodology, this study aims to answer the following questions: What factors influence the decision-making process of individuals in the United States who initially received a COVID-19 vaccine regarding whether to continue or not continue to receive the latest COVID-19 vaccinations? And to what extent have COVID-19 vaccination decisions been influenced by the presence or absence of symptoms of depression and/or anxiety?

## 2. Methods

### 2.1. Sampling and Recruitment

Participants were recruited from the Communities, Households, and SARS-CoV-2 Epidemiology (CHASING) COVID Cohort study. This is a fully online national prospective cohort study launched on 28 March 2020, with 6740 enrolled participants who complete follow-up assessments related to health and behaviors, symptoms of anxiety and depression, SARS-CoV-2 infection history, and COVID-19 vaccination status approximately monthly from March 2020 to July 2021 and then every quarter from September 2021 to December 2023. The community-based sample study included participants from diverse geographic and socio-demographic backgrounds, aged ≥ 18 years, and residing in the US or US territories. Comprehensive details regarding recruitment and follow-up of this parent study are presented elsewhere [19,20].

Recruitment for the present study focused on participants actively engaged in follow-up activities since July 2020 who completed a survey in June/July 2023 indicating receipt of at least one COVID-19 vaccine dose since they became available in December 2020. Additionally, a smaller subgroup of participants was selected to focus on individuals who had recently gotten a COVID-19 vaccine, consisting of individuals who reported receiving one since March 2023. To gather information regarding symptoms of anxiety and depression, participants completed validated questionnaires including the Generalized Anxiety Disorder 7-item (GAD-7) and the 8-item Patient Health Questionnaire (PHQ-8). Individuals scoring 10 or higher on the PHQ-8 or GAD-7 scales were classified as experiencing mental health symptoms. Responses were obtained from quarterly surveys conducted in June/July 2023 and April/May 2023.

The sample frame included a total of 1798 participants who reported receiving their last COVID-19 vaccine dose before 1 September 2022 and 249 participants who had reported receiving a COVID-19 vaccine dose on or after 1 March 2023. Respondents were contacted in batches. Individuals who met the inclusion criteria based on vaccination status were put into a bucket to be contacted at random. We aimed to achieve a quota of the following groups: five parents of children under five years of age, five people with symptoms of depression and anxiety, and five people at higher risk for severe COVID-19 outcomes based on age (65 years and older) and/or report of heart disease, diabetes, high blood pressure, chronic obstructive pulmonary disease (COPD), immunosuppression or an autoimmune disease, or current asthma, so that our findings could reflect on vaccination status and the intersection with parenthood, mental health status, and risk. Quota sampling was employed to ensure the inclusion of a diverse range of perspectives and experiences within the population. This approach provides insights into how various factors might influence people differently while effectively managing the limited resources of qualitative work, ensuring no single group is over-sampled or over represented, in line with the research objectives.

### 2.2. Materials

The interview guide was developed collaboratively among the research team and was structured into two main sections. The first one focused on the individual and their current, past, and projected experiences and feelings about the COVID-19 pandemic, the virus itself, and the vaccines, with questions such as “What are the most significant ways the COVID-19 pandemic has affected you and your family?” and “How did you decide whether or not to get a booster?”. This section also included probes for symptoms of anxiety and depression and probes that connected it to the second section of the interview guide, pertaining to information gathering. The second section delved into the health-related messages participants were exposed to, the sources of these messages, and how they determined which information to trust and act upon. Finally, a third supplemental section was designed for parents of young children and high-risk populations to explore differences in COVID-19 vaccine behavior related to these groups. Additionally, the research team held regular meetings throughout the interview process to evaluate the effectiveness of the questions. Based on these evaluations, they modified and added questions as necessary. It is noteworthy that after conducting 10 interviews, the team observed that most respondents mentioned having a personal doctor. Consequently, they added a specific question about this to the interview guide for the remaining 15 interviews. This iterative approach ensured that the interviews remained relevant and comprehensive.

Semi-structured interviews were carried out remotely during October, November, and December 2023 via telephone calls or Zoom. Participants received a USD 75 incentive in the form of a gift card. Verbal consent was obtained from all participants before recording the interviews, which were subsequently transcribed using the DataAgain transcription service.

### 2.3. Analysis

The transcripts were subjected to coding and thematic analysis framed within a grounded theory methodology. Grounded theory is a research methodology based on an inductive reasoning process; that is, hypotheses are developed from the data rather than data collection being a process of testing a pre-existing hypothesis [21]. This methodology aims to uncover the underlying processes and mechanisms that explain how and why certain phenomena occur, making it particularly useful for understanding behavior from the perspective of those involved, which, in this case, is behavior regarding COVID-19 vaccination in the endemic context of 2023.

In parallel, thematic analysis involves identifying, analyzing, and reporting patterns or themes within the data in a systematic and flexible way. In the context of grounded theory, this technique allowed for themes to emerge from the data and provide a structured framework for identifying patterns and relationships. Systematic cycles of inductive and deductive reasoning and verification allowed the research team to develop integrated theories of action within the specific epidemiological, social, and political contexts and connect it with existing literature [22].

Each transcript was reviewed by two independent reviewers. Each coder created an Excel sheet of topics that emerged from the transcript. Quotes were pulled out from the transcripts and then conceptually named by a coder. After each coder worked, the data were combined and organized using these Excel sheets; the co-coders reviewed similarities in their quotes and comments in order to group quotes that seemed to be about the same theme. This process was conducted by two independent coders. To ensure intercoder reliability, initial training and practice coding on smaller subsets of data with fewer interviews were implemented to identify areas of confusion and disagreement early on. Regular meetings, ongoing discussions, and feedback sessions were held with the larger group of researchers to maintain consistency and address any issues that arose. After this initial organization, the research team was able to identify ten main emerging categories: Health Behavior, General Vaccine Attitude, News Sources, Relationship to Institutions, Peer Network, Social Media, Barriers of Access, Mental Health, and Physical Health. Following the identification of these categories, a second codification process of each transcript was conducted in order to identify any and all quotes that fell under these categories but were not initially identified as such. Each category was then analyzed in order to identify their main characteristics. Posteriorly, additional information was added to the Excel table regarding occupation, geography, having a personal doctor, previously having COVID-19, having children, being part of at-risk population, having received a COVID-19 vaccine ever, and having received a COVID-19 vaccine booster. This information was derived from both survey data and qualitative interviews. In cases in which the information was contradictory, the data from the qualitative interviews took precedence. Each category was then analyzed by the research team in light of these stratification factors. The findings of this process are presented below.

## 3. Findings

### 3.1. Study Participants

Seventy-three people were invited to participate via email, of which thirty-eight did not respond or accept the invitation and nine accepted but did not show up to the interview. Hence, 25 interviews were conducted, 14 of which involved individuals who had been classified as experiencing recent symptoms of anxiety and depression according to the methodology previously described. Full demographic characteristics of participants based on information from the cohort study is provided in Table 1. Interviews lasted between 15 and 45 min and, on average, were 28 min in length. Of the 25 participants who were randomly selected within the quota stratum, 13 were women, 11 were men, and 1 identified as transgender. The sample included an equal number of White and Black participants (eight each), with five identifying as Hispanic, two as Asian/Pacific Islanders, and two as belonging to other racial/ethnic groups. Regarding age distribution, approximately half of the interviewees (12) were in the 18–34 age group, while six fell between the ages of 35 and 49, five were aged 50–64, and two were 65 years or older. In terms of income, the majority of participants (13) reported annual household incomes below USD 50,000. Eight participants earned between USD 50,000 and USD 100,000 annually, three reported incomes exceeding USD 100,000, and one participant’s annual income remained undisclosed. The participants were from 16 different states.

### 3.2. Vaccine Status

The qualitative interviews revealed that for some participants, vaccine status had changed since their last responses to the questionnaire. Given the introduction of a new vaccine in September 2023, it became more relevant to concentrate the analysis on these recent decisions rather than on vaccinations received before the summer season of 2023. Consequently, the final sample of 25 participants comprised 17 individuals who initially received a COVID-19 vaccine but had not remained up to date with subsequent vaccinations, who we refer to as ‘One-Shot Wonders’, and 8 individuals who had received all of the latest shots, hereafter ‘Booster Enthusiasts’.

The findings are presented in two parts, as follows: first, we present the reasons why individuals initially chose to get vaccinated; second, this is followed by an examination of the factors influencing their decisions to continue or discontinue receiving vaccinations. Notably, our analysis revealed no significant differences between One-Shot Wonders and Booster Enthusiasts in the first part, but very different discourses in this second part.

### 3.3. Why Did Individuals Get Vaccinated Initially?

Participants revealed five main nonexclusive reasons for receiving a COVID-19 vaccine initially. These are explained below, organized according to the frequency and importance of these reasons in participants’ accounts. In this analysis, importance is reflected by how participants portrayed specific reasons—how much they emphasized certain factors, how often they revisited them, and how they discussed these factors relative to others. Notably, in the initial stage, there were no conceptual differences between the reasons provided by participants who later received updated COVID-19 vaccines and those who did not.

1. Convincing information gathering—“I do my own research”

The primary reason for receiving an initial dose of a COVID-19 vaccine, mentioned by more than half of the participants, was their personal information gathering process which led them to believe getting the vaccine was the best choice. Participants cited their ability to form an opinion due to the consistency of information across various media sources. They described their reasoning by explaining how they formed their opinion about the vaccine, referencing a wide variety of information outlets. These included over 50 different news sources across the 25 participants, such as broadcasting channels, radio, print media, and news podcasts from local, state, national, foreign, and international organizations. Participants also mentioned obtaining information from local, state, national, foreign, and international health organizations, as well as from political figures, academic papers, communications from pharmaceutical companies, and social media influencers.

One participant exemplified the variety of sources they used to obtain information as follows:

“*I tend to want to research or not research, but like the teaching hospitals, like Emory, John Hopkins, Mayo Clinic, like I said, the CDC and the NIH, the British NHS, Australian and New Zealand NHS, Switzerland, and Sweden. I forget what those are called, the Nobel Institute I know had something going on, the UN Health site, WHO...*”—118408, One-Shot Wonder

The participants who identified this category as the primary reason for their decision to get vaccinated initially described their exposure to the information environment and how it contributed to their decision-making process, reflecting a context that is conducive to greater health literacy and empowerment in making informed choices about vaccination. The consistency of information led people to express they considered vaccination to be necessary to maintain their health and the health of people around them.

One participant stated the following:

“*I waited a little bit when it first came out, because I wasn’t too sure of it. I like to do my research on stuff before I get stuff or do anything. So once I did my research, I was like, regardless of what type of you know, negative stuff people were saying, I’d rather protect me and my family than not protect me and my family. So we went ahead and got it.*”—103929, Booster Enthusiast

In this regard, participants noted that during their process of information gathering, they encountered several specific messages that resonated with their personal situations, which ultimately served as a cue to action for receiving the vaccine.

One participant reflected on their underlying conditions, as follows:

“*Well, I have an immunocompromised system. So, I kept that in mind, thinking that my immune system is very low and that I could be susceptible to catching COVID unfortunately. So, I didn’t want to hesitate on getting it*”—102310, One-Shot Wonder

Similarly, others alluded to the importance of vaccination to protect people around them, as one participant explained the following:

“*I don’t want to pass it on to any of the vulnerable people that I interact with. So that’s a motivating factor.*”—119570, One-Shot Wonder

2. Institutional and Governmental Policies—The Carrot and the Stick

The second most frequently cited reason motivating vaccination was institutional and governmental policies. This encompassed both positive and negative incentives, such as financial rewards and paid time off, as well as restrictions or added burdens for those who remained unvaccinated.

One participant positively recounted the policies implemented by their employer:

“*I guess corporate brought it to my manager and then my manager sat all of us down and they said, “Well, if you get all four, which includes the two and then the two boosters, if you get all four, you get four days off and we’re paying you $40 for each one. So, the money was good at doing it. But just knowing that they cared enough that if we got it, if we got the vaccine and we had like side effects from it, we had those days off that we could recuperate from that. So, just that alone showed that they did care about it.*”—102310, One-Shot Wonder

Another participant expressed contempt around the established policies around vaccination:

“*Because we were forced into it. Remember if you wasn’t vaccinated, you wasn’t allowed in restaurants, you wasn’t allowed in many places you were to take care if you wasn’t vaccinated. I didn’t do it by choice […]*”—117439, One-Shot Wonder

3. Family Influences

Participants also mentioned being influenced by their family members to receive the vaccine. Some described them as positive influences from their family members, whom they trust and consult for guidance on vaccination decisions, as can be seen in the following remark:

“*I think I talked to my mom the most about it. She’s very for getting vaccines, you know, whatever vaccines are out there because she believes that, you know, vaccines protect us against diseases that can harm us. So I talk to her a lot when I’m like on the fence about whether or not I should get a vaccine for myself or my children*”—118862, One-Shot Wonder

In contrast, one participant reflected how family influences can be coercive, sparking actions through pressure, as follows:

“*My mom kind of made me. I mean it was that or I don’t have a place to live. So, it was a pretty easy decision to make.*”—118819, One-Shot Wonder

4. Participants also mentioned receiving recommendations from their own trusted physician to receive the vaccination. This included their PCPs, their children’s pediatrician, or a trusted doctor in their close network.

For instance, one participant noted the following:

“*So, mostly what I kinda get information and bring it to my doctor and try my doctor to explain it to me well. Try to make sure that whatever I find in the Internet, make sure it goes through my doctor so they can say.*”—103917, One-Shot Wonder

In this sample, at least 21 people had a personal doctor they said they trusted.

5. Finally, participants also alluded to their own personal experiences with COVID-19. This included people who had a serious case of COVID-19 that scared them, or had a family member that suffered the condition severely or passed away.

One participant mentioned the following:

“*...my mom actually did end up having COVID and was on a vent for about two weeks and made it through. That was probably the worst two weeks of my life when we weren’t sure if she was going to be able to make it through or not.*”—100614, Booster Enthusiast

Following discussions with both Booster Enthusiasts and One-Shot Wonders regarding their decision-making process and rationale for initially receiving a COVID-19 vaccine, it became evident that they all aligned with five nonexclusive categories. These include obtaining consistent information, which convinced them the vaccine was the best decision; feeling incentivized or pressured by institutional or government policies; being motivated or pressured by a family member; receiving physician recommendations; and/or being influenced by personal experiences with COVID-19. Moving forward, we focused on what had changed—what prompted these initial motivations for vaccination to become obsolete for a significant portion of the sample?

### 3.4. One-Shot Wonders: Why Did Individuals Stop Receiving COVID-19 Vaccines?

Though conveyed in different ways, One-Shot Wonders consistently expressed doubt about the necessity of the vaccine at the time of the interviews. This doubt was informed by different pieces of information they had read or heard and was manifested in the ways in which they talked about COVID-19 disease and vaccine information. Some perceived COVID-19 to no longer be threatening, and others perceived the efficacy of the vaccine to be limited, among other things. Across the specific comments of this group, almost all participants had encountered an information environment that no longer presented the vaccine as necessary to maintain their health and the health of the people around them. This can be translated into four different factors.

1. “Covid is gone”

The most common conception explained by participants was their perceived absence of COVID-19 and how that affected their vaccine attitude. When asked why they did not receive the latest vaccine, one person noted the following:

“*Okay, because I feel like COVID-19 is gone and it might not be serious like it was before around 2020. It might not be that serious anymore, so that’s why.*” —119291

Along this same line, some people were simply unaware of the latest vaccinations, as follows:

“*No, it’s been kinda—we haven’t really heard anything about it. Maybe it’s just the period, I don’t know.*”—117533

2. “There’s a different strand”

This information environment also led some people to develop doubts about the efficacy of the vaccines and the delivery of information about vaccines and the virus’ evolution. As one person noted the following:

“*Honestly, I have mixed feelings about it because I’ve heard so many different things. Oh, it’s not going to work or there’s a different strand. They don’t have the vaccination for this particular strand, this new strand that they said that came out. I have mixed feelings about it because I, like I said, I’ve heard so many different things. I just don’t know whether it’ll be effective or not.*”—102310

3. “Nobody ever said anything about a second round of it”

Simultaneously, the evolving information landscape implies that certain influencers or entities that previously advocated, incentivized, or pressured individuals to receive vaccines may no longer be as prominent. This encompasses family members, peers, healthcare providers, and institutions that were once strong proponents of vaccination.

One person referred to the policies instituted by their children’s school as follows:

“*So, after that their school, nobody ever said anything about a second round of it.*”—117533

Another person referred to their doctor, as follows:

“*I think I’ve tried asking my doctors about hey, should I be getting this booster kind of like the flu shot? And I’ve gotten a mixed bag of yes and no and it’s up to you and I’m like okay, but I need a medical professional’s advice. I understand you’re trying not to be disrespectful if I am anti-vax, but now I’m kind of like. I feel now I’m like I don’t know, you’re being too vague about how often I should be getting the vaccines.*”—118364

4. Lack of consistent information

Participants also noted a lack of consistency and contradictions in the messaging they received, which led the following participant to distrust all sources:

“*So with all that context, I’ve chosen not to continue with the vaccine series. I could re-evaluate that decision, but that’s what’s informing me is. So I think that the political environment in the US has really diminished my trust in the polarized, the tribalism, the polarization, the propaganda has really clouded my ability to trust what I’m hearing in the US.*”—116945

The inconsistent public health communications left room for possible misconceptions or assumptions, as noted by another participant, as follows:

“*I think it seems like maybe COVID is doing the thing that all good viruses do, where they become more contagious but less deadly to their hosts over time.*”—119570

Finally, it’s worth noting that among the seventeen participants in this category, only one individual indicated they remained in a similar mindset to when they initially received their first COVID-19 shot, and cited logistical concerns as their primary reason for not receiving the latest vaccine. They stated the following:

“*I’m in a limited transportation situation. And if I could just jump in a car and go get one, I’d do that. But I have to, it literally takes me a week to coordinate a doctor’s appointment before I even make the doctor’s appointment. You know what I’m saying? And where I live, there ain’t no such thing as Uber, because I’m way out in the country.*”—118408

### 3.5. Booster Enthusiasts: What about the Fully Vaxxed?

After examining the rationale behind the decision of 17 participants not to pursue staying up to date on their COVID-19 vaccinations, we contrasted their perspectives with the eight people who did remain up to date with their vaccinations. We analyzed the initial list of five reasons provided and assessed which factors continued to influence their decisions.

Our analysis revealed that, out of the five initial reasons—(1) convincing information gathering, (2) institutional policies, (3) family pressure, (4) doctors’ recommendations, and (5) individual experiences—only the first two influenced their decisions to remain up to date with COVID-19 vaccinations. Upon closer examination, we observed that almost every participant who continued to receive vaccinations had strong ties to either the healthcare or education sectors, allowing them to remain connected to particular sources and types of information and policies regarding COVID-19. At least five of the participants in this group mentioned working directly in healthcare-related fields (e.g., emergency department worker, public health officer, PhD student in neuroscience and psychology, pharmacist, and special education therapist) and highlighted how their professions compelled them to stay abreast of the latest vaccination guidelines.

Some participants noted their own personal role in disseminating accurate information within their network, as follows:

“*I know that I’m also the source of a lot of that information for a lot of my friends [...] If I can verify something or debunk some information that I see on social media, I do go out of my way to share the truth about it, like to fact check with my peers and my colleagues and my friends and my family members.*”—100614

Similarly, another participant expressed their role within their peer group, as follows:

“*I’ve been a very early adopter of getting the vaccine. So I probably among my circle of friends and family I’m the one that always seems to get it first.*”—103431

Another person expressed their commitment to advocating for vaccines and related it to their field of work, as follows:

“*But yeah, I was very positive about vaccines right from the get-go, because I am at least science adjacent, if not a scientist myself [...] So I really I’m an active proponent of those and I’ve advocated vaccines, sometimes successfully to all of my friends and colleagues and family members*”—103929

Another participant spoke about the importance of vaccines for their own health and the feeling of exposure they have at work, as follows:

“*I’ve just always gotten it, because I’ve had the flu before, but it’s been many years, it was not fun. And the fact that having asthma and not being able to breathe already and I grew up very sickly. So, and working around kids is my reason if they are coughing or germs everywhere. So, I want extra protection.*”—105361

Overall, these participants emphasized their commitment to staying informed about health- and vaccine-related information. Their exposure to this information continues to influence their belief in the necessity of vaccination for their own health and the well-being of those around them.

### 3.6. Mental Health—Depression and Anxiety

The analysis also sought to address the following question: to what extent are COVID-19 vaccination decisions influenced by the presence or absence of symptoms of depression and/or anxiety?

The sample comprised 14 individuals who had scored 10 or higher on the Generalized Anxiety Disorder (GAD-7) and/or the Patient Health Questionnaire (PHQ-8) (see Section 2.Methods), as well as 11 individuals who did not exhibit such symptoms on at least one of the two most recent quantitative surveys conducted by the research team. However, the interviews revealed no clear pattern between the presence of symptoms of depression and anxiety and vaccination status. This may reflect that symptoms were not present at the time in which the qualitative interviews were conducted or inconsistent for participants. Participants with these symptoms were distributed evenly between One-Shot Wonders and Booster Enthusiasts, and across all emerging themes in relation to the reasons behind vaccine decisions.

Only one exception emerged in the case of a participant who shared that their agoraphobia and anxiety hindered them from leaving the house, highlighting logistical and practical barriers that impeded their vaccination efforts, as follows:

“*I’m agoraphobic. I have panic attacks. So like I said, the pandemic itself was not a real hardship on me, but getting, but now if I wanted to get out and get information or talk to somebody about it, that’s complicated*”—118408, One-Shot Wonder

On another note, it is worth noting that only 6 of the 14 participants that showed symptoms of depression and anxiety in the quantitative data acknowledged or recognized a heightened state of anxiety or depression when discussing their mental health in the interviews. While analyzing the data, it became evident that many people were experiencing distress without necessarily associating it with a state warranting concern.

As one person noted the following:

“*I think health wise it did, even now I would have to say I have a hard time having the motivation to leave the house and go work out, as opposed to before the pandemic. If I had to go work out I would go work out, it wasn’t, I don’t know, I feel it’s difficult now and I don’t know why?*”—118364, One-Shot Wonder

Another person narrated their current hardships, as follows:

“*Certainly there was a whole lot more worry and thoughts about well, just having to make different day to day living plans and certainly the isolation and the loss of a lot of friendships. So, yeah, and then trying to be supportive of my closest friends in my little bubble who are going through the same thing. And to top that off and having those couple losses of family members during that time and not being able to gather in a way that would have provided some emotional support. So, yeah, that way, and then the loneliness and not only dealing with my own but trying to be supportive of my adult son as well who is experiencing similar things.*”—103431, Booster Enthusiast

## 4. Discussion

This study illustrates the significant evolution of the COVID-19 vaccination landscape over the last few years. Accounts from both Booster Enthusiasts and One-Shot Wonders depict a shift from an environment conducive to vaccination across personal, interpersonal, community, organizational, and policy spheres to one characterized by a notable absence of the continued need and efficacy of COVID-19 vaccines. This has led to reduced concern regarding COVID-19 infection, severe illness, or complications due to long COVID, limited perceived benefits of the vaccine, and fewer cues to action. Notably, only those institutionally and intellectually tied to the healthcare and education sectors continued to be immersed in an information environment that promoted continued COVID-19 vaccination that may have led them to stay up to date with the vaccines.

Our findings highlight the role of the information environment in shaping respondents’ COVID-19 vaccination decisions across message content and messengers. Consistency of information proved to be the primary driver for initial vaccination among all participants. They heard about the COVID-19 vaccine in so many places and spaces, and they took action, collectively mentioning over 50 different media outlets that provided information about the COVID-19 vaccine. However, when discussing the present day, most participants struggled to recall hearing about the vaccine in the media, creating a striking discrepancy in information environments between One-Shot Wonders and Booster Enthusiasts. While One-Shot Wonders described a lack of awareness of a new vaccine and a perception that COVID-19 is no longer a concern, the majority of Booster Enthusiasts described how they were immersed in a healthcare or education environment that tends to stay up to date with the latest vaccination guidelines.

Our findings also suggest a notable shift in which sources of information are influential. Historically, researchers have focused on the effect of family and personal experiences on an individual’s decision-making processes [23,24]. Despite the family setting being typically considered one of the most influential on a person’s health and social beliefs, family influences ranked only third among the reasons cited by participants for initial vaccination, shadowed by people’s reliance on their own information gathering, as well as institutional policies. Moreover, family influences showed little to no continuity or consistency over time, not appearing as a reason to continue vaccination as seen in the discourses provided by Booster Enthusiasts. This is consistent with recent literature suggesting that the majority of people use traditional media to obtain information about the COVID-19 vaccine, and this use increases the likelihood of vaccination [25].

Other factors could also be contributing to this shift, including changing family structures. Participants described a wide array of family sizes, arrangements, and configurations, and also narrated transitions in their family composition occurring over the last few years. This diversity in family structures challenges models that center around a “traditional” family, where family influences are consistent and one-directional [23]. Some studies have assessed how the roles of people within the family have changed in recent decades, where children and young adults, for example, are now taking on more active roles as agents promoting their own health, rather than passive recipients of guidance from older family members [26]. Moreover, increasing individualism in society encourages people to make decisions autonomously, moving away from traditional family-centric decision-making paradigms.

These results are consistent with the way participants talked about their own personal experiences with COVID-19. Evidence has shown how personal experience often challenges or overrides scientific consensus and professional insights when it comes to people’s health [24], and experiences with severe symptoms of COVID-19 have been associated with a greater perception of susceptibility to and fear of the virus [27]. Nevertheless, our findings found personal experiences to be the least mentioned factor in people’s decision to get vaccinated initially, and personal experiences with COVID-19 did not come up in Booster Enthusiasts’ accounts as a relevant reason to continue up to date with vaccines. Even a person who described their mother’s encounter with COVID-19 as being the “worst weeks of their life” did not regard the vaccine to be necessary for their own health nowadays. This speaks directly to people’s perception of the severity of the virus, and how threatened they currently feel by COVID-19.

Physician recommendations have also been examined and found to be influential on decision making. Among the 25 interviewees, 21 mentioned having a trusted personal physician, and in their accounts, doctor’s recommendations played a significant role in their decision to get vaccinated initially. However, notably, Booster Enthusiasts did not mention physician recommendations as a factor in their decision to continue receiving vaccines. Moreover, some One-Shot Wonders expressed confusion and mixed messages about recent conversations with their physicians regarding the updated COVID-19 vaccine. While physician recommendations have been found to reduce vaccine hesitancy [28] and were instrumental in motivating vaccinations during the initial stages, there is limited evidence regarding physicians’ recent efforts to promote booster shots and why they may not continue to do so. Studies in other countries have explored physicians’ burnout and exhaustion due to the pandemic, and the decreased perception of their level of responsibility and influence on vaccine uptake [29]. Others have highlighted their need for additional support and resources to identify unvaccinated individuals, as well as the time constraints and tensions of engaging in conversations regarding vaccines [29], though more studies would be needed to explore this phenomenon in US physicians, reports have shown record-breaking levels of physician burnout since the pandemic [30,31]. Providing support and additional resources may show potential to improve proactive engagement and conversations surrounding the COVID-19 vaccine.

Although only one person cited logistical reasons for not getting the latest vaccine, it is important to note the changes in the accessibility landscape since the initial COVID-19 vaccine rollout. During 2021 and 2022, localities across the country offered multiple ways to get vaccinated, including mass vaccination sites, mobile vaccination vans, and accommodations for people with special needs or disabilities [32,33]. Additionally, the vaccine was provided at no cost. Currently, individuals must navigate through their insurance providers to receive the updated COVID-19 vaccines, and most would have to schedule an appointment and/or visit a pharmacy or health facility. This shift could be impacting more people’s ability to get vaccinated, and improving accessibility could be beneficial for the upcoming COVID-19 vaccine rollout.

In terms of socioeconomic status, it is notable that the majority of participants in our sample fell into the low-income bracket (13 out of 25). The qualitative data indicated that some participants experienced significant income changes during the pandemic, such as job losses or securing better employment after graduation. However, specific income details were not directly asked during the interviews, so the income data presented reflect the most current information available. Despite these nuances, the distribution of vaccine uptake among different income brackets aligns relatively well with existing literature. Specifically, in the low-income group, 9 out of 13 participants were categorized as One-Shot Wonders. In the USD 50 K–100 K bracket, 6 out of 8 participants were One-Shot Wonders, whereas in the USD 100 K + bracket, 2 out of 3 participants were Booster Enthusiasts. Although these data are not intended to be generalizable, it supports the trend observed in the literature that higher income is associated with higher uptake of vaccine boosters [34].

Interestingly, further exploration of the qualitative data reveals that the trend was more closely related to participants’ ties with healthcare and education rather than strictly their income levels. Among the four Booster Enthusiasts in the <50K income bracket, there was a healthcare worker, a healthcare student, and a teacher. In contrast, One-Shot Wonders included a hotel worker, a store clerk, homemakers, and individuals who reported being unemployed. In the next income bracket, the two Booster Enthusiasts were a special education therapist and a public health officer. These results underscore that while income is an important factor, qualitative research provides deeper insights into the experiences and contexts behind the numbers. This leads us to highlight the value of our sample. Although it is concentrated among lower-income groups and has less representation from higher-income groups, research has indicated that this demographic is often more prone to vaccine hesitancy and tends to be underrepresented in research studies and the media [34,35]. Therefore, documenting their perspectives is particularly valuable and provides important context for understanding vaccine uptake and elaborating targeted public health messaging interventions.

Considering all of the abovementioned factors, this study makes valuable contributions to the existing literature by examining both individual and macrolevel factors that could influence vaccine hesitancy and behavior. It is one of the few studies that use a grounded theory approach to understand people’s subjective experiences and perceptions of COVID-19 in the current scenario and gain a deeper understanding of theoretical factors and variables. In terms of behavioral science constructs, it is clear that people’s perceptions of their susceptibility to a disease are effective cues to action; however, this perception is influenced by the information environment to which they are exposed. At the same time, the perceived benefit of vaccination is greater when people can relate to the messages they hear and feel personally identified with them, as seen with individuals who discussed having an immunocompromised system or working/living with vulnerable populations. Although barriers to preventive actions were not a major factor in this study, they should not be disregarded for future pandemic preparedness. In terms of theories that focus on macro-level factors, it was evident that both public policy and organizational influences were significant cues to action during the initial rounds of vaccination as seen by people who got vaccinated because of their jobs or mobility restrictions; however, these were not supported by convincing information gathering, thus limiting their continuity and impact over time. Additionally, interpersonal and community-level influences, such as family pressure and doctor recommendations, seem to occupy a space between policy- and individual-level cues to action. By strengthening public health messaging and introducing policies to support physician’s ability to do vaccine outreach to their patients, they have the potential to enhance COVID-19 booster uptake.

Only a year since the WHO declared the end of the COVID-19 global health emergency, the lack of awareness and perceived susceptibility to the threat of COVID-19 should be a concern for public health and emergency preparedness efforts. The absence of direct messaging opens the door to misinformation and misconceptions, as seen in participants who believed “Covid is gone”, described their confusion regarding the current strands, lack of efficacy related to the updated vaccines, and expressed disbelief of current information outlets. Given the changing landscape of COVID-19 information, public health messaging and outreach efforts need to align with people’s current attention, disposition, and capacity. This includes emphasizing that while COVID-19 is no longer in a pandemic stage, the virus remains a significant concern in an endemic context. Furthermore, it is crucial to provide clear information on the effectiveness of vaccines against different strains of the virus, reassuring the public about the benefits of getting vaccinated with updated boosters. All in all, it is crucial to remind people that the virus did not follow a natural course toward disappearance or to become “the good virus”: it is only controlled if we take the necessary steps to control it.

Finally, the relationship between symptoms of depression and anxiety and COVID-19 vaccination was not particularly salient and did not rise to the level of thematic saturation to suggest this was a pattern among respondents. On the other hand, it became notable to the research team that several participants discussed a normalization of symptoms of depression and anxiety during the pandemic that warrants concern and further research. Our findings highlight the continued need to address mental-health-related stigma and raise awareness of symptoms of anxiety and depression and the benefit of evidence-based mental health care. This is especially important in periods such as the COVID-19 pandemic, when intense hardships and avoiding engaging in activities have become normalized. Having said that, the broader body of evidence related to the effect of symptoms of depression and anxiety on vaccination still remains limited and equivocal. Additional research on this topic is needed.

## 5. Conclusions

Our study employed a grounded theory approach to explore the factors influencing COVID-19 vaccine decision making among individuals in the United States and examined the effect of symptoms of depression and anxiety on these decisions. The findings illustrate how the COVID-19 landscape has evolved over the past few years, transitioning from an environment that actively promoted vaccination across personal, interpersonal, community, organizational, and policy spheres to one characterized by a notable absence of COVID-19 conversations or messaging. This shift has led to reduced worry about infection and severe illness, diminished perceived benefits of the vaccine, and fewer cues to action. Interestingly, only individuals institutionally and intellectually connected to the healthcare and education sectors remained immersed in an information environment that encouraged ongoing COVID-19 vaccination, likely influencing them to stay up to date with their vaccines. Our study also highlights that while consistency of information was the primary driver for initial vaccination among all participants, influences such as family, personal experiences, and physician recommendations, though important initially, did not maintain their influence over time. Finally, the relationship between symptoms of depression and anxiety and COVID-19 vaccination was not particularly salient for most respondents, and logistical barriers to accessing the vaccine were not commonly cited as limitations despite the changing landscape in provision and accessibility in recent vaccine rollouts. Overall, the implications of this study underscore the importance of accurate and timely public health messaging that adapts to individuals’ dispositions and misconceptions. Emphasizing the dynamic nature of public health communication and developing mechanisms to effectively reach populations with changing needs and concerns are crucial for future public health initiatives.

## 6. Strengths and Limitations

This study employed a grounded theory approach to explore participants’ decision-making processes regarding COVID-19 vaccine uptake. This technique is particularly useful for understanding social processes, actions, and interactions in a way that is more likely to be applicable since it is based directly on participants’ real-life accounts, instead of relying on pre-existing theories or data. However, it does mean that results are highly contextual and specific to participants’ environments, and although the study included individuals from across the United States, this specificity should be considered when thinking of policy and research implications. In this particular case, a significant portion of the sample was associated with the healthcare and education sectors, which could suggest sample bias and may influence responses in ways that are not representative of the US population. Nevertheless, qualitative research does not aim for broad representativeness but rather seeks to provide a deep, nuanced understanding of specific phenomena, and our findings offer valuable insights into people’s opinions and feelings regarding COVID-19 vaccines. Additionally, the interviews were conducted in the fall of 2023, and the endemic scenario of COVID-19 has continued to evolve since then. While the drivers identified for vaccination are likely still relevant, new concerns may have emerged that warrant consideration. Future qualitative research is encouraged to continue enhancing our understanding and refining policies related to COVID-19 vaccination.

The sampling methods used in this study have both strengths and limitations. Recruiting from the CHASING COVID Cohort study, an online prospective cohort, may introduce selection bias since participants engaged in an online longitudinal study about COVID-19 might differ from those who are not. However, this approach has the advantage of involving participants who already have an established relationship with the research institution, potentially leading to more open and honest responses. While the sample encompasses a range of geographic and socio-demographic backgrounds, the smaller subgroup of recent vaccine recipients may not be as diverse or representative. Despite this, their interviews offered valuable insights into the experiences of this specific group. Additionally, quota sampling, while useful for ensuring diverse perspectives, had limitations due to the small quotas (e.g., only five participants per group), which in some cases did provide sufficient data for the research team to draw interesting and robust conclusions about these subpopulations.

## Figures and Tables

**Table 1:** Participant Sociodemographic Characteristics.

	Number
Gender	
Women	13
Men	11
Nonbinary/transgender/other	1
Race/Ethnicity	
Black	8
White	8
Hispanic	5
Asian/Pacific Islander NH	2
Other NH	2
Age	
18–34	12
35–49	6
50–64	5
65+	2
Income	
<50 K	13
50–100 K	8
100 k+	3
Unknown	1
State (N of participants)	Texas (4), New York (3), Ohio (2), Illinois (2), North Carolina (2), Nevada (2), New Jersey (1), Washington DC (1), Delaware (1), Florida (1), Georgia (1), Indiana (1), Michigan (1), Oklahoma (1), Utah (1), Washington (1)

## Data Availability

The raw data supporting the conclusions of this article will be made available by the authors on request.
